# Comparison of End-Tidal Carbon Dioxide Values in ICU Patients with and Without In-Hospital Cardiac Arrest

**DOI:** 10.3390/biomedicines13020412

**Published:** 2025-02-08

**Authors:** Kaitlyn Dalton, Jeffrey J. Mucksavage, Dustin R. Fraidenburg, Kevin He, James Chang, Maria Panlilio-Villanueva, Tianxiu Wang, Scott T. Benken

**Affiliations:** 1 Ascension Seton Medical Center, Austin, TX 78705, USA; kdalton104@gmail.com; 2 Department of Pharmacy Practice, University of Illinois Chicago College of Pharmacy, Chicago, IL 60612, USA; jmuck@uic.edu; 3 Division of Pulmonary, Critical Care, Sleep, and Allergy, Department of Medicine, University of Illinois Chicago College of Medicine, Chicago, IL 60612, USA; dfraiden@uic.edu; 4 Department of Pharmacy, Rush University Medicine Center, Chicago, IL 60612, USA; kevinhe825@gmail.com; 5 Regulatory Advertising and Promotion, Astellas Pharma US, Northbrook, IL 60062, USA; jchang67@uic.edu; 6 Division of Nursing, University of Illinois Hospital and Health Sciences System, Chicago, IL 60612, USA; mpanli1@uic.edu; 7 Center for Research on Health Care, Department of Medicine, University of Pittsburgh, Pittsburgh, PA 15260, USA; tiw93@pitt.edu

**Keywords:** in-hospital cardiac arrest, capnography, resuscitation, monitoring, intensive care unit, cardiovascular, prevention

## Abstract

**Objective**: The purpose of this study was to evaluate the utility of end-tidal carbon dioxide (ETCO_2_) values as a predictive marker of in-hospital cardiac arrest (IHCA). This was achieved by comparing the trends of ETCO_2_ values in mechanically ventilated ICU patients that experienced an IHCA versus patients that did not. **Methods**: A single-center, retrospective, observational, and comparative cohort study at an academic medical center. Mechanically ventilated adults in the ICU who received continuous ETCO_2_ monitoring were included. Patients who were transferred to our facility already intubated, experienced an out-of-hospital cardiac arrest, or had a do-not-resuscitate order were excluded. Extracted data points included demographics, comorbidities, vitals, labs, and outcomes. Patients were grouped into IHCA and non-IHCA cohorts, and the trends of ETCO_2_ values were compared at multiple time points for 48 h before the IHCA or after intubation (time zero) for the groups, respectively. An ROC curve was constructed to determine the predictive value of ETCO_2_ for IHCA. **Results**: A total of 207 patients were included, of which 104 (50.2%) had an IHCA and 103 (49.8%) did not. There were no differences in the mean SOFA scores at the initiation of mechanical ventilation (8.5 vs. 7.6). The ETCO_2_ values were decreased in the IHCA cohort, and significantly different at each time point analyzed from 300 min until immediately prior to the arrest (*p* < 0.001). The ETCO_2_ values were a mean of 20.0 mmHg in the IHCA cohort at the index time vs. 34.7 mmHg in the non-IHCA cohort (*p* < 0.001). The ROC analysis demonstrated moderate reliability, with an AUC = 0.687 (*p* < 0.0001, 95% CI 0.613–0.761) and with an ETCO_2_ of less than 23 mmHg, demonstrating a 67% sensitivity and a 71% specificity, as well as a 70% PPV for predicting the IHCA from our sample. **Conclusions**: Patients typically have rapid clinical deteriorations prior to cardiac arrest, and monitoring ETCO_2_ is easily achieved at the bedside while aiding in clinical decision making. The ETCO_2_ values in our study were significantly decreased in the IHCA cohort prior to cardiac arrest compared to the stable values in those that did not experience an IHCA, indicating that ETCO_2_ monitoring may have utility in predicting cardiac arrest. Further study is warranted to evaluate if predictive models utilizing ETCO_2_ can be constructed to predict IHCAs in mechanically ventilated ICU patients.

## 1. Introduction

It is estimated that 292,000 adults in the United States suffer in-hospital cardiac arrests (IHCAs) annually, and approximately 20% survive until hospital discharge [1,2,3]. The Get with the Guidelines (GWTG) Resuscitation Registry (formerly the National Registry of Cardiopulmonary Resuscitation) was initiated to focus on the prevention of cardiac arrests through quality improvement efforts and research [4,5]. Overall, patients undergoing IHCAs have better outcomes than patients arresting in the out-of-hospital setting, likely due to the utilization of rapid response teams and the immediate initiation of basic life support [2,3,4]. Since most cardiac arrests in the inpatient setting are secondary to acute respiratory compromise, patients experiencing IHCAs will typically have a progressive deterioration prior to the event [2,6,7]. This supports the notion that early surveillance, detection, and prevention may be of substantial benefit to improve clinical outcomes for IHCA. Guidelines state that early-warning sign systems (EWSSs) using the electronic medical record (EMR) and weighted scoring tools may be utilized; however, this is listed as a conditional recommendation due to the lack of robust data validating any one particular tool in the intensive care unit (ICU) [8]. Our group aimed at identifying the early markers of cardiorespiratory instability that can potentially be used to impact the care of the critically ill and redirect dangerous clinical trajectories.

End-tidal CO_2_ is the amount of CO_2_ detected at the end of exhalation, with normal values ranging from 35 to 45 mmHg [9,10,11]. End-tidal CO2 monitoring is currently utilized to assess the quality of chest compressions and to predict the return of spontaneous circulation (ROSC) during cardiac arrest, to confirm the placement of an advanced airway such as an endotracheal tube, and to evaluate the ventilation status during general anesthesia [12]. Within the past two decades, continuous ETCO_2_ monitoring has become more routine in the ICU, particularly in mechanically ventilated patients [9,10,11]. Under normal conditions, it is a non-invasive means of measuring patients’ alveolar ventilation status, since it closely correlates with the partial pressure of arterial carbon dioxide (PaCO_2_) [10,13]. Data also suggest that there is a correlation between ETCO_2_ values and cardiac output [11,14]. This may imply that decreasing ETCO_2_ values could represent an additional objective data point obtained at the bedside for impending decreasing cardiac output and, ultimately, cardiac arrest. To our knowledge, limited literature exists evaluating the utility of ETCO_2_ predicting cardiac arrest.

A pilot cohort study of 104 patients with IHCA observed that mean ETCO_2_ values significantly decreased immediately prior to IHCA compared to most comparison time points, affirming the need for comparison analyses and modeling explorations with another cohort [15]. Though promising, this pilot study was limited by the lack of a comparator group, leading to hesitation in drawing firm conclusions. Our current study builds on this prior work and we hypothesize that, in mechanically ventilated ICU patients, there will be a significant and clinically detectable difference in ETCO_2_ values leading up to IHCA compared to patients that do not experience an IHCA. The purpose of this study was to compare those values to evaluate if ETCO_2_ monitoring has a role in predicting IHCA.

## 2. Materials and Methods

### 2.1. Study Design

**Figure 1:**
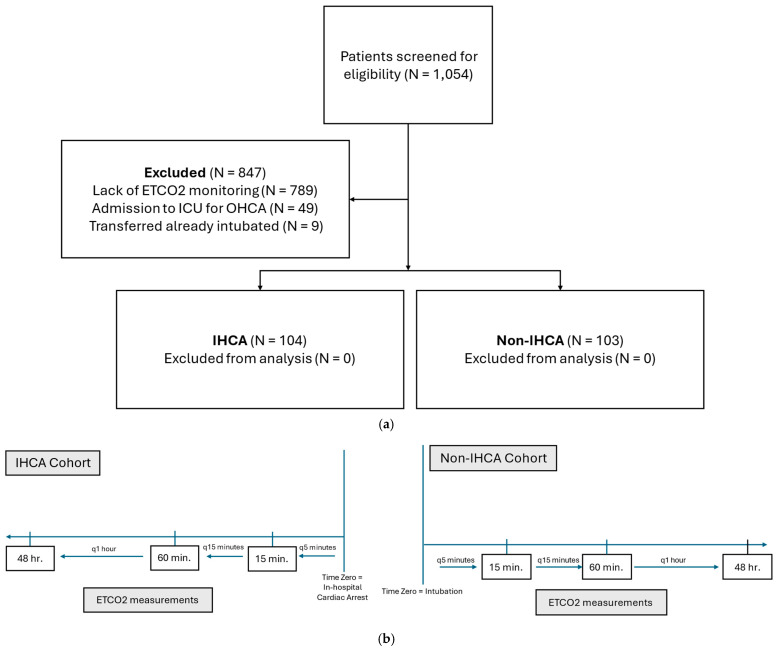
(**a**). **CONSORT diagram.** Patients screened, reasons for exclusion, and cohort allocation are shown above. Abbreviations: DNR = do not resuscitate; ETCO_2_ = end-tidal carbon dioxide; ICU = intensive care unit; IHCA = in-hospital cardiac arrest; OHCA = out-of-hospital cardiac arrest. (**b**). **ETCO_2_ measurement schematic.** Patients that suffered from an IHCA had their ETCO_2_ values captured from the previous 48 h of the IHCA. The interval of measurement was every 5 min for the 15 min before the arrest, then every 15 min for the 60 min before the arrest, then every 1 h for the 48 h before the arrest. The non-IHCA cohort had their ETCO_2_ values captured for 48 h after their intubation. The interval of measurement was every 5 min for the 15 min after the intubation, every 15 min for the 60 min after the intubation, and then every 1 h for the 48 h after the intubation. Abbreviations: IHCA = in-hospital cardiac arrest; ETCO_2_ = end-tidal carbon dioxide.

This study was a single-center, retrospective, observational, and comparative cohort study at an academic medical center comparing the trends of ETCO_2_ values in mechanically ventilated ICU patients that had an IHCA versus patients that did not (Figure 1a). In patients that experienced an IHCA, individual ETCO_2_ values were collected for a total duration of 48 h before the IHCA. For patients that did not have an IHCA (i.e., the non-IHCA cohort), the index time (time “zero”) was when the patient became mechanically ventilated, and data were collected for 48 h after that intubation. Individual ETCO_2_ values were recorded every 5 min for the first 15 min preceding the IHCA or after the intubation of the non-IHCA group. They were then recorded every 15 min for the first hour and then hourly through 48 h of evaluation from the index time in both groups (Figure 1b). This index time for the non-IHCA cohort was chosen for the following 3 reasons: (1) the average duration of mechanical ventilation in our ICU at the time of this investigation was 48 h, (2) there was limited availability of ETCO_2_ monitoring in patients who were not mechanically ventilated at our institution at the time of investigation, and (3), in multiple analyses, the duration of mechanical ventilation was associated with mortality and, as such, limiting the “non-IHCA” group to 48 h could potentially falsely eliminate increased risk of mortality with a longer duration of mechanical ventilation [16,17,18]. All of the patients included from both cohorts were analyzed during the same time period to ensure that the practice patterns were similar. The study was approved by the Institutional Review Board (IRB) at the University of Illinois at Chicago (IRB Protocol:2019-1112).

### 2.2. Study Patients

Adult patients greater than 18 years of age that were admitted to the ICU and receiving mechanical ventilation for at least 48 h with continuous ETCO_2_ monitoring were eligible to be included in the study population. Patients in the IHCA cohort experienced a documented IHCA and had Advanced Cardiac Life Support (ACLS) performed. The study reviewed records of hospitalized patients for 3 consecutive years. Patients were excluded if they were transferred to our facility while already intubated or if they were admitted to the ICU after undergoing an out-of-hospital cardiac arrest (OHCA). Patients on whom care was withdrawn or those with a do-not-resuscitate (DNR) order were also excluded. These patients in the IHCA cohort were identified previously in our pilot investigation [15] from a list of ICD-9 and ICD-10 codes for IHCA. The non-IHCA cohort was identified using a list of ICD-9 and ICD-10 codes for mechanical ventilation in the ICU with manual cross-referencing for inclusion using consecutive patients during the same time period until the target number of patients was reached.

### 2.3. Data Collection and Study Outcomes

Demographic data of age, race, and sex were recorded for each patient. Past medical history (PMH), including the presence of arrhythmias, coronary artery disease (CAD), and/or chronic kidney disease (CKD), were collected at ICU admission from the admission note. Clinical variables, including an arterial blood gas (ABG) and laboratory values such as serum chemistry and lactic acid, sequential organ failure assessment (SOFA) scores, and the use of vasopressors and/or antibiotics, were also collected immediately prior to the IHCA for the IHCA cohort and at the time the patient became mechanically ventilated (i.e., the index time, or time zero) in the non-IHCA cohort. For patients that experienced an IHCA, variables of the initial rhythm and the achievement of ROSC (defined as a sustained heart rate and rhythm, and blood pressure leading to the cessation of resuscitation efforts) were recorded. The primary comparison was ETCO_2_ values at all time points from the index time (i.e., time zero) to 48 h between patients suffering an IHCA and those that did not. The secondary comparison was an ICU and hospital length of stay (LOS), mortality, and discharge disposition (defined as being alive at the end of hospitalization and discharged to either home, a long-term care facility, acute rehab, or hospice) between groups. Data collected was conducted manually by three investigators with cross-auditing to ensure consistency.

### 2.4. Statistical Analysis

Chi-squared or Fisher’s Exact tests were employed to detect the differences in categorical variables. For continuous variables, an assessment of normality was performed utilizing the Shapiro–Wilk test. For normally and non-normally distributed variables, the *t*-test and the Wilcoxon Rank-sum test were used, respectively. A two-tailed alpha level of 0.05 was considered statistically significant. For the primary outcome, the ETCO_2_ values were compared between cohorts at each time point utilizing two-sample *t*-tests. Since the same groups of subjects were compared multiple times, the alpha value was adjusted using the Bonferroni correction. A *p* < 0.0025 indicated statistical significance. Sample size calculations were based on identifying a 20% change in the ETCO_2_ between groups, as it was felt that this would represent a clinically meaningful change; with an 80% power and an alpha error of 0.05, it was determined that a minimum of 100 subjects per group would be required. The time point cutoff for the retrospective review was based on this sample size calculation, and all of the subjects meeting the inclusion criteria through this time point were included, which resulted in just over 100 subjects in each group. Additionally, a Receiving Operator Characteristic (ROC) curve was constructed to determine the predictive value of the minimum ETCO_2_ and the maximum ETCO_2_ values in predicting IHCAs in ICU patients.

## 3. Results

Of the 1054 mechanically ventilated ICU patients during the specified time, 207 mechanically ventilated patients met the inclusion criteria for this study (Figure 1). A total of 104 patients (50.2%) experienced an IHCA, while 103 patients (49.8%) did not. The most common reason for patient exclusion criteria was a lack of ETCO_2_ monitoring (93.2%). Of note, routine ETCO_2_ monitoring was not in place at our institution in the early part of the study period. The mean (±SD) age of patients included was 57.1 (±15.0) years. Included patients were primarily Black (88, 42.5%) and White (47, 22.7%) races, and were in the medical ICU (191 [92.3%] MICU; Table 1). All of the patients, totaling 103 (100%), in the IHCA cohort were being cared for under the MICU service at the time of the IHCA. More patients in the IHCA cohort had a PMH of arrhythmias (41.3% vs. 9.7%, *p* < 0.001) and CAD (28.8% vs. 13.6%, *p* < 0.007) at baseline, and a greater use of vasopressors at the index time (i.e., time zero) (71.2% vs. 40.8%, *p* < 0.001). There were no statistically significant differences in age, sex, CKD, SOFA scores, or antibiotic use between the IHCA and non-IHCA cohorts. Patients that experienced an IHCA had a statistically significant lower median (IQR) pH (*p* < 0.002), higher serum potassium (<0.001), lower serum calcium (*p* = 0.042), and higher lactic acid (*p* < 0.001) compared to patients that did not have an IHCA (Table 2). A total of 72 (69.2%) patients that experienced an IHCA had pulseless electrical activity (PEA) as the initial rhythm and 14 (13.5%) patients presented with asystole. Only seven (6.7%) patients had either ventricular tachycardia or ventricular fibrillation that caused an IHCA.

**Figure 2:**
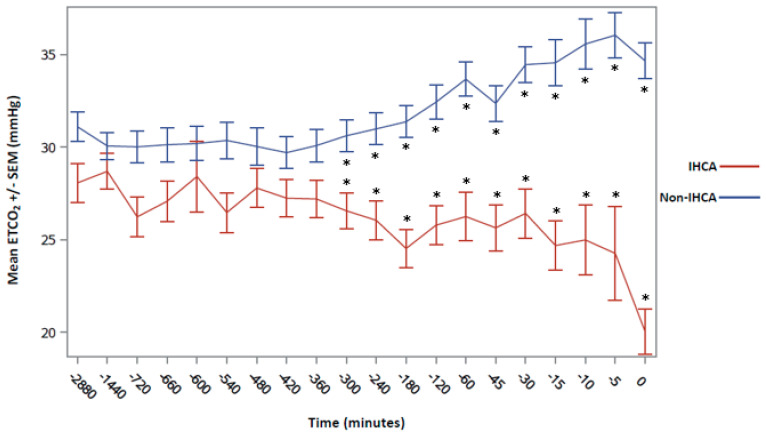
**Mean ETCO_2_ values leading up to the IHCA compared to the non-IHCA reference time points.** Mean ETCO_2_ values in patients that experienced an IHCA compared to patients that did not experience cardiac arrest. Time “zero” in the IHCA cohort was immediately prior to cardiac arrest. In the non-IHCA cohort, time “zero” was when the patient became mechanically ventilated, and data were recorded for 48 h after the initiation of mechanical ventilation. Abbreviations: ETCO_2_ = end-tidal carbon dioxide; IHCA = in-hospital cardiac arrest; SEM = standard error mean. ETCO_2_ values are reported as mean ± SD. (* = statistically significant with an alpha level set at 0.0025.)

**Figure 3:**
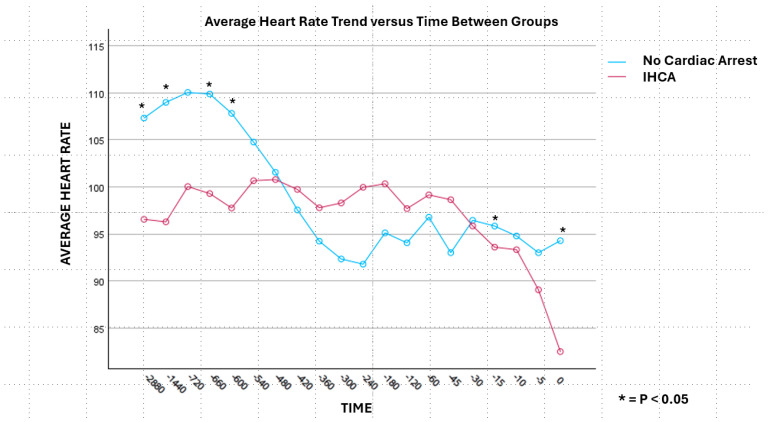
Average HR values in beats per minute in patients who experienced an IHCA compared to patients who did not experience cardiac arrest. Time “zero” in the IHCA cohort was immediately before cardiac arrest. In the non-IHCA cohort, time “zero” was when the patient became mechanically ventilated, and data were recorded for 48 h after the initiation of mechanical ventilation. Abbreviations: HR = heart rate; IHCA = in-hospital cardiac arrest. HR values are reported as mean. (* = statistically significant with alpha level set at 0.05.)

**Figure 4:**
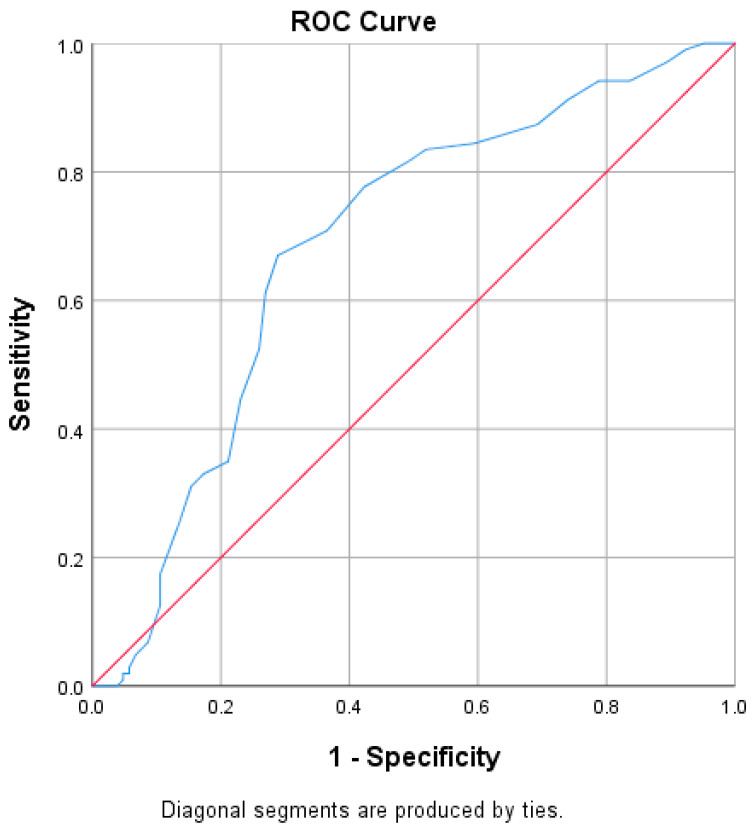
**Receiver Operating Curve (ROC) for minimum ETCO_2_ values leading up to the IHCA.** The red line is the random model line for the ROC curve and the blue line represents the observed model. Area under the curve (AUC) = 0.687, *p* < 0.0001, and 95% CI 0.613–0.761. If an ETCO_2_ of 23 mmHg is chosen, the subsequent sensitivity = 67% and the specificity = 71%, with a PPV of 70% for predicting the IHCA.

For the primary outcome, the ETCO_2_ values were decreased in the IHCA cohort at each time point from 48 h (2880 min) until immediately prior to the cardiac arrest compared to the non-IHCA cohort (Figure 2). The difference in ETCO_2_ values beginning at 300 min until immediately prior to the arrest were statistically significant at each time point when utilizing an adjusted a priori-defined alpha (*p* < 0.0025). Immediately prior to the arrest, the ETCO_2_ values were a mean (±SD) of 20.0 mmHg (±10.13) in the IHCA cohort compared to 34.7 mmHg (±9.4) in the non-IHCA cohort (*p* < 0.001). Interestingly, the trend in heart rates (HRs) between groups did not follow a similar pattern to ETCO_2_, except for the time point nearest to the cardiac arrest (Figure 3). An ROC analysis for the minimum ETCO_2_ demonstrated moderate reliability, with an AUC = 0.687 (*p* < 0.0001, 95% CI 0.613–0.761, Figure 4) and with an ETCO_2_ of less than 23 mmHg, demonstrating a 67% sensitivity and a 71% specificity for predicting an IHCA. The median [IQR] ICU LOS was 5 [2,3,4,5,6,7,8,9,10,11] vs. 11 [7,8,9,10,11,12,13,14,15,16,17,18,19,20,21] days, while the median [IQR] hospital LOS was 7 [3,4,5,6,7,8,9,10,11,12,13,14,15,16] vs. 15 [8,9,10,11,12,13,14,15,16,17,18,19,20,21,22,23,24,25,26] days in the IHCA and non-IHCA cohorts, respectively (*p* < 0.001). The outcomes in this study were poor, with only 4 patients in the IHCA cohort surviving to hospital discharge compared with 14 patients in the non-IHCA cohort, indicating mortality rates of 92.6% vs. 86.4%, respectively (*p* = 0.014). One patient was discharged home, while three patients went to acute rehab upon discharge in the surviving IHCA cohort. Among the 14 surviving patients in the cohort that did not have an IHCA, 9 of them were discharged to a long-term care facility (LTAC), while 1 patient went to acute rehab and hospice.

A stepwise, conditional multivariable logistic regression model was constructed utilizing the variables collected in this analysis. Categorical variables for model consideration included vasopressor therapy, coronary artery disease, arrhythmia past medical history, antibiotic therapy at the time of inclusion, chronic kidney disease, and sex. Continuous variables for model consideration included ETCO_2_ values from various time points, HR values from various time points, the ICU length of stay, and the hospital length of stay. The ETCO_2_ at 0–4 min and HR from 0–4 min were the strongest predictors of an IHCA. Every 1 mmHg decrease in ETCO_2_ was associated with a 1.3 times greater likelihood of an IHCA (*p* = 0.004). Every 1 beat per min decrease in HR was associated with a 1.06 times greater likelihood of an IHCA (*p* = 0.007). No variables were statistically associated with the development of an IHCA in this model.

**Table 1:** Baseline characteristics.

	IHCA (n = 104)	Non-IHCA (n = 103)	*p*-Value
Age, median years (IQR)	61.5 (50–70)	58.0 (49–66)	0.068
Race, n (%)			<0.001
Black	53 (51)	35 (34)	
White	19 (18.3)	28 (27.2)	
Hispanic/Latino	1 (1)	16 (15.5)	
Unknown/Other	31 (29.8)	24 (23.3)	
Males, n (%)	62 (59.6)	55 (53.4)	0.367
Past medical history, n (%)			
CAD	30 (28.8)	14 (13.6)	0.007
Arrhythmia	43 (41.3)	10 (9.7)	<0.001
CKD, n (%) ^#^	25 (24.0)	15 (14.6)	0.113
Stage II	1 (4)	0	0.317
Stage III	3 (12)	2 (13.3)	0.317
Stage IV	0	2 (13.3)	0.317
Stage V/dialysis	21 (84)	11 (73.3)	0.392
SOFA score, median (IQR) ^	7.0 (4–10)	9.0 (5–11)	0.126
Vasopressor therapy, n (%)	74 (71.2)	42 (40.8)	<0.001
Antibiotics, n (%)	85 (81.7)	93 (90.3)	0.076

# CKD was classified and staged per KDIGO criteria: Stage I kidney damage with normal or increased GFR (≥90 mL/min/1.73 m^2^); Stage II kidney damage with a mild decrease in GFR (60–89 mL/min/1.73 m^2^); Stage III moderate decrease in GFR (30–59 mL/min/1.73 m^2^); Stage IV severe decrease in GFR (15–29 mL/min/1.73 m^2^); Stage V kidney failure defined as GFR < 15 mL/min/1.73 m^2^ or dialysis [27]. ^ SOFA score measures the degree of organ dysfunction in six organ systems (respiratory, coagulatory, liver, cardiovascular, renal, and neurologic) with ranges from 0 to 20; the higher numbers indicate more severe organ dysfunction [28]. Abbreviations: CAD = coronary artery disease; SOFA = sequential organ failure assessment.

**Table 2:** Clinical characteristics.

	IHCA (n = 104)	Non-IHCA (n = 103)	*p*-Value
Arterial blood gas, median (IQR)			
pH	7.29 (7.12–7.38)	7.33 (7.23–7.44)	0.002
pCO_2_, mmHg	37 (29–47)	40 (30–51)	0.053
pO_2_, mmHg	90 (72–117)	89 (67–122)	0.436
Laboratory values, median (IQR)			
Potassium, mmol/L	4.5 (3.9–5.1)	4.1 (3.7–4.6)	<0.001
Calcium, mg/dL	7.9 (7.3–8.5)	8.1 (7.5–8.7)	0.042
Magnesium, mg/dL	2.1 (1.9–2.4)	2.0 (1.8–2.2)	0.017
Blood urea nitrogen, mg/dL	37 (22–52)	27 (16–50)	0.051
Serum creatinine, mg/dL	2.3 (1.4–3.6)	1.6 (0.9–3.0)	0.005
Glucose, mg/dL	130 (100–172)	128 (107–177)	0.270
Lactic acid, mmol/L	4.1 (2.0–9.8)	2.4 (1.4–3.9)	<0.001

In the IHCA cohort, arterial blood gas and laboratory values were collected before the IHCA occurred at the time nearest the event, but not during the IHCA. In the non-IHCA cohort, values were recorded at the initiation of mechanical ventilation. *p* < 0.05 indicates statistical significance.

## 4. Discussion

To our knowledge, this is the first study in a mechanically ventilated cohort of ICU patients that evaluated the trends of ETCO_2_ values in patients who experienced an IHCA compared to patients who did not. Patients in the IHCA cohort had a statistically and clinically significant decline in their ETCO_2_ values leading up to the cardiac arrest compared to the ETCO_2_ values obtained in the non-IHCA cohort, which remained relatively stable for the 48 h of mechanical ventilation during our study window. The relatively consistent values of ETCO_2_ in the non-IHCA cohort and the early and significant decrease in ETCO_2_ values in the time leading up to the IHCA may lend this variable to aid in the early detection of IHCAs. Additionally, once the ETCO_2_ fell below 23 mmHg, there was an adequate specificity and a 70% PPV for predicting the IHCA, which may lend that threshold as a key indicator for the treatment team to evaluate and/or intervene if possible. The ICU and hospital LOS values were significantly longer in the non-IHCA cohort, likely due to more patients in the IHCA cohort experiencing mortality earlier on in their hospital stay. Additionally, there was high mortality in both cohorts, and poor overall outcomes and disposition.

The findings in our study were clinically relevant, as ETCO_2_ monitoring has become a common practice in the ICU. As noted above, ETCO_2_ monitoring is a non-invasive way to measure a patient’s alveolar ventilation status and is correlated with cardiac output [9,11,13,14]. Several studies have observed ETCO_2_ values as a predictor of mortality and achieved the ROSC during the performance of CPR for patients who experienced IHCAs [19,20,21]. Still, our group has focused on evaluating ETCO_2_ as a predictive tool before IHCAs. In using ETCO_2_ during resuscitation, studies found that average ETCO_2_ values greater than 10 mmHg were associated with a higher likelihood of achieving the ROSC. While helpful, it is known that the outcomes of IHCAs are generally poor, so it is paramount to turn our attention to the pre-event clinical detection [1,2,3]. Our study observed a rapid decline in ETCO_2_ values leading up to the IHCA. The deterioration in ETCO_2_ occurred earlier than HR changes in patients before the event. Interestingly, there was a period when both ETCO_2_ and HR were “stable”, followed by the change in ETCO_2_ and then the change in HR. Perhaps there is an opportunity to evaluate the “uncoupling” of these stable values to help further predict deterioration. Regardless, given our findings and the feasibility of monitoring ETCO_2_ in mechanically ventilated ICU patients, this is an additional objective data point that should be routinely monitored to alert clinicians of potential impending clinical deterioration.

The early detection of deteriorating clinical status has been found to improve clinical outcomes in the hospital setting. The Modified Early Warning Score (MEWS) is a physiologic scoring system that can be incorporated into EMRs to alert clinicians that a rapid bedside assessment of a patient’s clinical status is necessary [22]. The scoring tool provides information on derangements in vital signs, including systolic blood pressure, respiratory rate, heart rate, temperature, and mental status [22]. Data support the use of the MEWS in general ward patients for predicting cardiac arrest, mortality, and requiring a higher level of care [22,23,24]. The MEWS tool has been studied, but has shown conflicting results, in the critically ill patient population [25,26,29]. A prospective study in critically ill patients observed that a MEWS ≥ 6 was an independent predictor of mortality in the ICU [30]. In a critically ill cohort of 925 patients presenting to the emergency department, a cutoff of MEWS ≥ 3 predicted cardiac arrest with a sensitivity of 74.4%, a specificity of 54.2%, and a negative predictive value of 97.8% [26]. Other studies in critically ill patients presenting to the emergency department found that composite MEWS scores did not perform well in predicting poor patient outcomes [25,29]. ETCO_2_ values are a potential variable missing from the MEWS tool. Given its correlation with cardiac output and the findings of our study, the addition of ETCO_2_ values to the MEWS tool may aid in a better prediction of cardiac arrest. Prediction models utilizing the MEWS have suggested that this tool can predict deterioration six hours prior to a clinically significant event [31]. Similarly, our current study shows that ETCO_2_ decline became significantly different from the control group about five hours prior to the cardiac arrest episode. This would indicate that this variable may add additional power to already utilized clinical prognostic calculators, but it also represents a biomarker with early clinical significance, such that poor outcomes are unlikely to be inevitable and there may be opportunity to alter the clinical trajectory. Blankush et al. conducted a study implementing a monitoring system using an automated device to capture the MEWS while incorporating ETCO_2_ values into the score to identify post-surgical non-ICU patients at risk for adverse events earlier in their hospital course [32]. Providers received automated pages for elevated MEWS scores, abnormal ETCO_2_ levels, or oxygen desaturations below 85%; however, there were many false alarms from falsely recorded vital signs. ETCO_2_ was added to an elevated MEWS score in 42 patients. While 50% of these included a falsely abnormal ETCO_2_ value, there were 21 instances where staff would not have been alerted to impending clinical deterioration if ETCO_2_ had not been added to the MEWS score. This suggests that adding ETCO_2_ values to the MEWS tool could capture more patients with impending clinical deterioration, although it is unknown if that would be the case in a cohort of ICU patients. More studies are needed to evaluate if ETCO_2_ monitoring can be incorporated into a modified MEWS tool to predict cardiac arrest in the ICU.

Interestingly, our ICHA cohort showed a slightly lower pH in the laboratory assessments before intubation. It is unclear how this may have influenced care, resulted from care, and/or affected ETCO_2_. Hypocapnia is known to have detrimental effects on cerebral perfusion and vascular tone, particularly after a cardiac arrest [33]. While the standard institutional approach is to target respiratory compensation without over-correction to respiratory alkalosis, there may have been individual instances of over-correction, and the impact of this in developing an IHCA is unknown. Our data show that these instances, if present, are likely rare, given that the median pCO_2_ was well above the severe hypocapnia range (<30 mmHg) in both groups. Future studies, mainly when designing an intervention, should need to carefully monitor for both hypocapnia and hypercapnia, both of which can have essential impacts on critical care outcomes.

There were several limitations in this study. Due to the retrospective study design, some of the data points were unavailable in the EMR. We believe we have adequate data points to capture the clinical backdrop and clinical course of patients for this analysis. Additionally, 74.8% of the screened population were excluded from the study if they did not have continuous ETCO_2_ monitoring so to ensure an adequate sample for data analysis. While this was a limitation during the study period, our institution has now moved to routine ETCO_2_ monitoring in all mechanically ventilated, and many other, patients, making the applicability of these data very much clinically relevant. Three of the research personnel completed data extraction from the EMR manually due to the inability to utilize automation. We utilized cross-auditing to ensure fidelity in data extraction and interpretation. Given the retrospective nature, it was challenging to determine the best comparative groups for this study. We chose to include those with ETCO_2_ monitoring available in both groups receiving mechanical ventilation simultaneously. Given the optional nature of this monitoring at the time at our institution, this biased the inclusion of patients that were mechanically ventilated with a high severity of illness, as demonstrated by the limited survival in both groups of patients. Fortunately, the ETCO_2_ values remained somewhat consistent throughout the study period in our control group, which served nicely to demonstrate the opportunity for ETCO_2_ changes to serve as a predictive variable for IHCA in ICU patients. There is a lack of widespread availability of ETCO_2_ values in non-mechanically ventilated patients. Still, it is known that they also suffer from IHCAs [3], making these data potentially applicable to all ICU patients regardless of the ventilation status. Lastly, there could have been inherent differences in the patient groups, leading to differences in the ETCO_2_ values that were not captured in the baseline data collection. The study could not capture dynamic changes in care due to the specific ETCO_2_ levels, either from a change in ventilator settings or changes to medical management. Thus, we cannot determine if some subjects had effective interventions that averted an ICHA event. The patients did have similar SOFA scores, rates and degrees of renal dysfunction, and the requirement of mechanical ventilation, which would make that less likely, but will remain a limitation given the retrospective nature. As a single-center study, we are encouraged by the findings, and future goals include validation in a prospective design as well as the potential implementation of a care algorithm designed to intervene when dynamic changes in ETCO_2_ are encountered.

## 5. Conclusions

Patients who experienced an IHCA in our mechanically ventilated cohort of ICU patients, compared to those without IHCA, had a statistically and clinically significant decrease in ETCO_2_ values leading up to the arrest. This work indicates that ETCO_2_ monitoring may have utility in predicting cardiac arrest, and further studies are necessary to evaluate if models utilizing ETCO_2_ can be constructed to predict an IHCA in mechanically ventilated ICU patients prospectively.

## Data Availability

Data sharing is not available for this study.
